# Trends in cervical cancer mortality in China from 1989 to 2018: an age-period-cohort study and Joinpoint analysis

**DOI:** 10.1186/s12889-021-11401-8

**Published:** 2021-07-06

**Authors:** Menghan Guo, Juan Xu, Jiayue Du

**Affiliations:** 1 School of Medicine and Health Management, Tongji Medical College of Huazhong University of Science & Technology, Wuhan, 430030 Hubei Province China; 2 Hubei Provincial Research Center for Health Technology Assessment, Wuhan, 430030 Hubei Province China; 3 Centre for Social Research in Health, University of New South Wales, Sydney, Australia

**Keywords:** Cervical cancer, Age-period-cohort model, Joinpoint regression, Mortality, China

## Abstract

**Background:**

Worldwide, cervical cancer is the second-most-common malignancy of the female reproductive system. Due to its large population, China accounted for 11.9% of cervical cancer deaths, and 12.3% of global cervical cancer DALYs in 2017. In 2009, China launched a nationwide screening program, yet mortality from cervical cancer has shown an upward trend in recent years. The aim of this study was to explore factors affecting cervical cancer mortality rates in China, and contribute to their future reduction.

**Methods:**

In this descriptive study, a Joinpoint regression analysis and age-period-cohort (APC) model based on the intrinsic estimator (IE) algorithm were utilized. Data from the period 1989–2018 were extracted from the International Agency for Research on Cancer (IARC) Database of WHO (1989–2000) and China Health Statistical Yearbook database (2002–2018).

**Results:**

Our study found mortality from cervical cancer to have initially declined, but increase thereafter over the entire observation period in both rural and urban China. The influence of age, period and cohort effect on the mortality rate had statistical significance. The effect of age increased with years, becoming a contributing factor in women aged over 45 years countrywide. Conversely, the cohort effect became a protective factor for women born after 1938 in urban areas, and for women born after 1958 in rural areas. The period effect was relatively less impactful.

**Conclusions:**

The study indicates that organized cervical screening projects facilitated the identification of potential patients, or patients with comorbidities. Correspondingly, mortality was found to increase with incidence, particularly among elderly women, indicating that newly diagnosed patients were at an advanced stage of cervical cancer, or were not receiving appropriate treatment. Therefore, the coverage of cervical cancer screening should be improved, and women’s health awareness promoted. Early diagnosis and treatment is critical to reduce the disease burden and improve outcomes.

## Highlights

1. Results showed an upward trend in cervical cancer mortality rates in both rural and urban China in recent years. We noticed a positive age effect but a negative cohort effect, which allowed for effective public intervention measures to be designed and targeted for these specific population.
2. Promotion of screening strategies in China may be required.
3. Timely treatment following a positive result from screening is necessary to prevent advanced cervical cancer and potentially death, and ultimately reduce mortality rates.
4. To make this study more informative and weight its scientific evidence, the Joinpoint model and the age-period-cohort model are used to provide more detailed analysis.
5. The data in our study covered 31 provinces in China’s mainland and collected from Vital Registration in China, to ensure the data was of good quality.

## Background

Cervical cancer is the second most common gynaecological cancer in the world [1]. In 2020, according to the International Agency for Research on Cancer (IARC), there were about 604,127 new cases of cervical cancer and 341,831 new deaths from cervical cancer worldwide, accounting for 6.5 and 7.7% of new cases and new deaths of women worldwide, respectively [2]. As the fourth leading cause of cancer deaths among women globally [2], cervical cancer represents a major threat to women’s health. Since the introduction of cervical cancer screening and the human papillomavirus (HPV) vaccine, the incidence and mortality rates of cervical cancer have shown a long-term downward trend worldwide [3]. In China, however, cervical cancer mortality rates have not yet fallen to similarly ideal levels. Due to its large population, China accounted for 11.9% of global cervical cancer deaths, and 12.3% of cervical cancer DALYs in 2017 [4]. Given the country’s vast landmass and uneven levels of economic development, there are significant disparities in the rates of the occurrence of and death from cervical cancer across regions [5].

Cervical cancer screening is recognized as an effective intervention to prevent the occurrence of advanced disease and death from cervical cancer. The World Health Organization (WHO) has stated that long-term and systematic cervical cancer screening can achieve up to 93% reduction in cervical cancer mortality [6]. To compensate for the imbalances caused by regional differences in its development, China launched the National Cervical Cancer Screening Program in Rural Areas (NCCSPRA) in 2009, concentrating on less developed regions. A total of 221 rural districts in 31 provinces were selected as project counties. Free screening services were provided for registered rural women aged 35–64 years with informed consent. Pap smears are the main screening method used whereas analysis of cervical cells is based on the Bethesda System (TBS) or Pap Grading Report. In resource-scarce areas, Visual Inspection with Acetic Acid Strain (VIA) or Visual Inspection with Logol’s Iodine (VILI) is applied. This project has also had some positive impacts in parts of urban China. Since 2009, an increased number of locally organized screening projects have been supported by municipal finance [7]. Yet the mortality rates of cervical cancer among urban and rural women in China have appeared to increase in recent years. Why have cervical cancer mortality rates continued to rise following the introduction of screening programs? Do screening programs in China need to be improved? Preventing the mortality of cervical cancer is, therefore, a topical issue in China that is worth discussing.

This study therefore aims to explore the factors affecting cervical cancer mortality, and help prevent future cases of invasive cervical cancer in China. Based on internal differences in China’s economic development, this study distinguishes between rural and urban areas of the country to better compare mortality trends and examine the underlying reasons. Furthermore, to make this study more informative and weight its scientific evidence, the Joinpoint model and the age-period-cohort model are used to provide more detailed analysis.

## Method

### Data sources

Cervical cancer mortality data were extracted from the IARC Database of the WHO (1989–2000) and China Health Statistical Yearbook database (2002–2018). Because mortality data for 2001 was not attainable from either database, we calculated the averages from the surrounding calendar years to compensate. Corresponding Chinese population data were extracted from the China Statistical Yearbook database. All data is freely available and open for public perusal. The data in our study covered 31 provinces in China’s mainland and collected from Vital Registration in China, to ensure the data was of good quality [8, 9].

Our study collected the cervical cancer case data of women aged 20–84 years, because cases occurring under the age of 20 years are rare and cases over the age of 84 years might implicate a variety of other causes of death. Cervical cancer mortality data were stratified into thirteen 5-year age groups (20–24, 25–29, 30–34, …, 80–84) and six 5-year periods (1989–1993, 1994–1998, …, 2014–2018). With confluence of age groups and periods, we calculated thirteen corresponding 5-year birth cohorts (1909–1913, 1914–1918, …, 1994–1998) [10]. The age-standardized mortality rate (ASMR) was calculated by direct standardization using the world standard population released by the WHO [11]. We attached detailed data in Tables 1 and 2 in Appendix.

### Statistical analysis

Trends in cervical cancer mortality were assessed by the Joinpoint Regression Program (JRP), using Joinpoint Regression Software (4.6.0.0 version, 2018) issued by the United States National Cancer Institute. JRP examines changes by detecting whether any differences between each segment of annual percent change (APC) are significant. A ‘Joinpoint’ will appear on a log scale if a statistically significant change occurs in trends. The significance assays use a Monte Carlo permutation method. Statistical significance is set at *P* < 0.05.

The age-period-cohort model is based on the Poisson distribution, and describes the variance, or “effect estimate” of in trends of cervical cancer mortality according to age, period, and cohort effects [12, 13]. That is, if the estimate of age, period and cohort effects is greater than zero, it represents a risk factor. Otherwise, it is a protective factor. To resolve the linear relationships among age, period, and cohort, the Intrinsic Estimator (IE) method proposed by Yang and Fu was applied [14–16]. Yang states that the IE method is a robust approach to resolving this problem, owing to its wide range of applications and because it doesn’t require a conditional assumption. The Akaike Information Criterion (AIC) and the Bayesian Information Criterion (BIC) were applied to observe how well the model fit [17]. The statistical analysis was made using Stata/SE 14 software (StataCorp, 2015).

## Results

### Overall trends of cervical cancer mortality rates

**Fig. 1:**
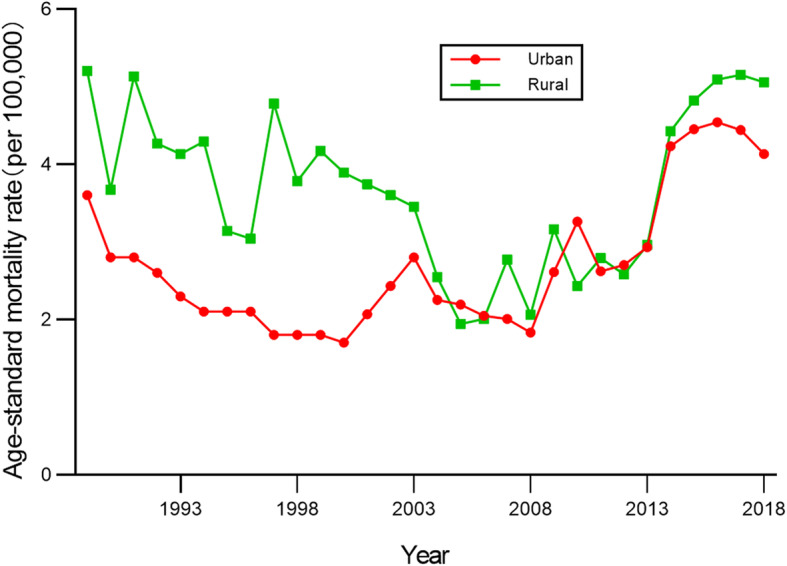
Age-standardized mortality rates of cervical cancer ASMR in urban and rural China, 1989–2018

**Fig. 2:**
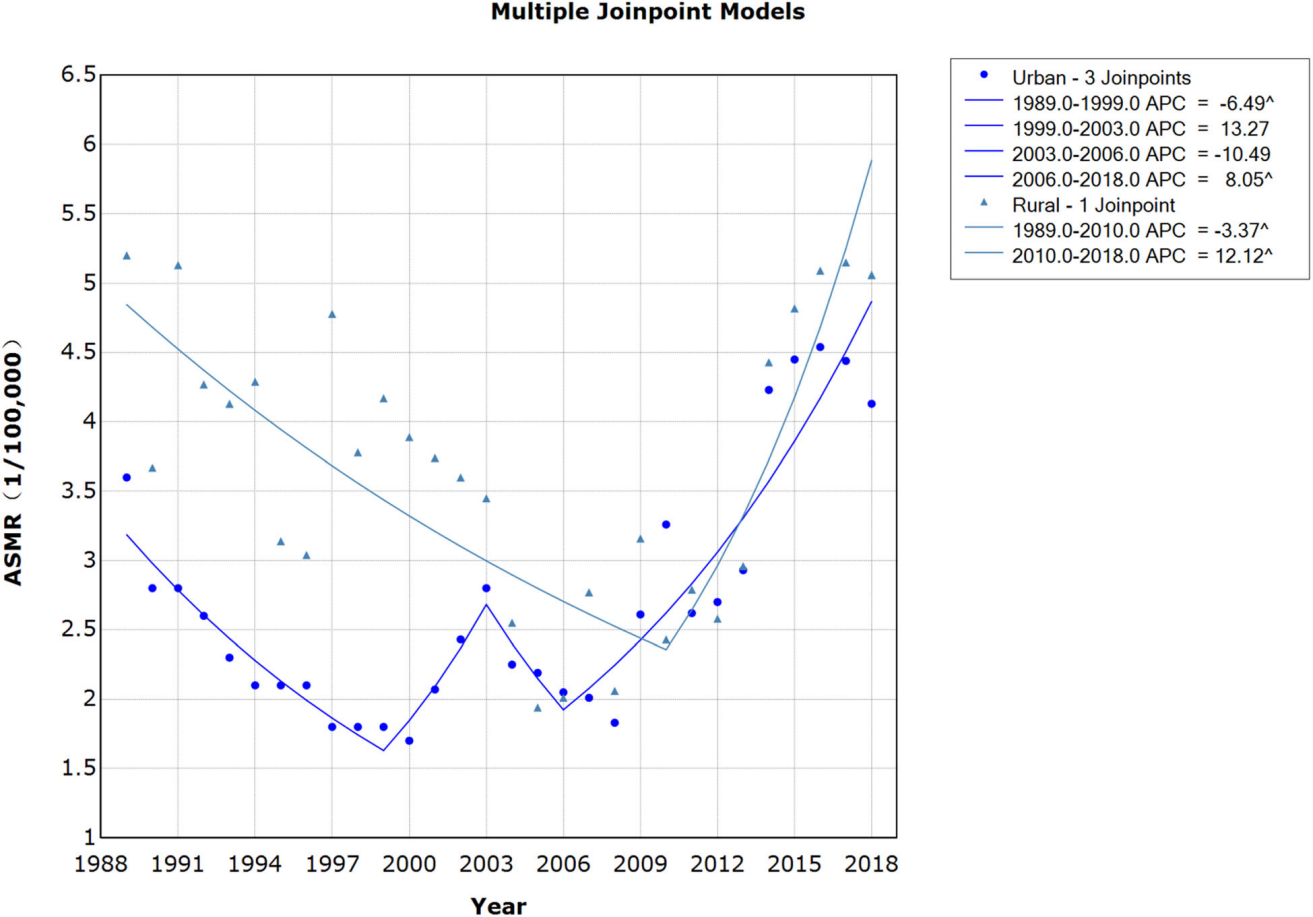
Joinpoint regression in cervical cancer ASMR in urban and rural China, 1989–2018

ASMR for cervical cancer in urban and rural China during the period 1989–2018 are presented in Figs. 1 and 2. As they show, the ASMR in both urban and rural areas initially fell (APC: − 6.49% vs − 3.37%), and then rose (APC: 8.05% vs 12.12%). In general, the ASMR in rural China were higher than it in urban. Following Joinpoint regression, one seemingly ‘V’-shaped Joinpoint was found in the rural trend. And three Joinpoints were found in the urban trend, resembling a “W” shape..

### Age-specific cervical cancer mortality rates by time period

**Fig. 3:**
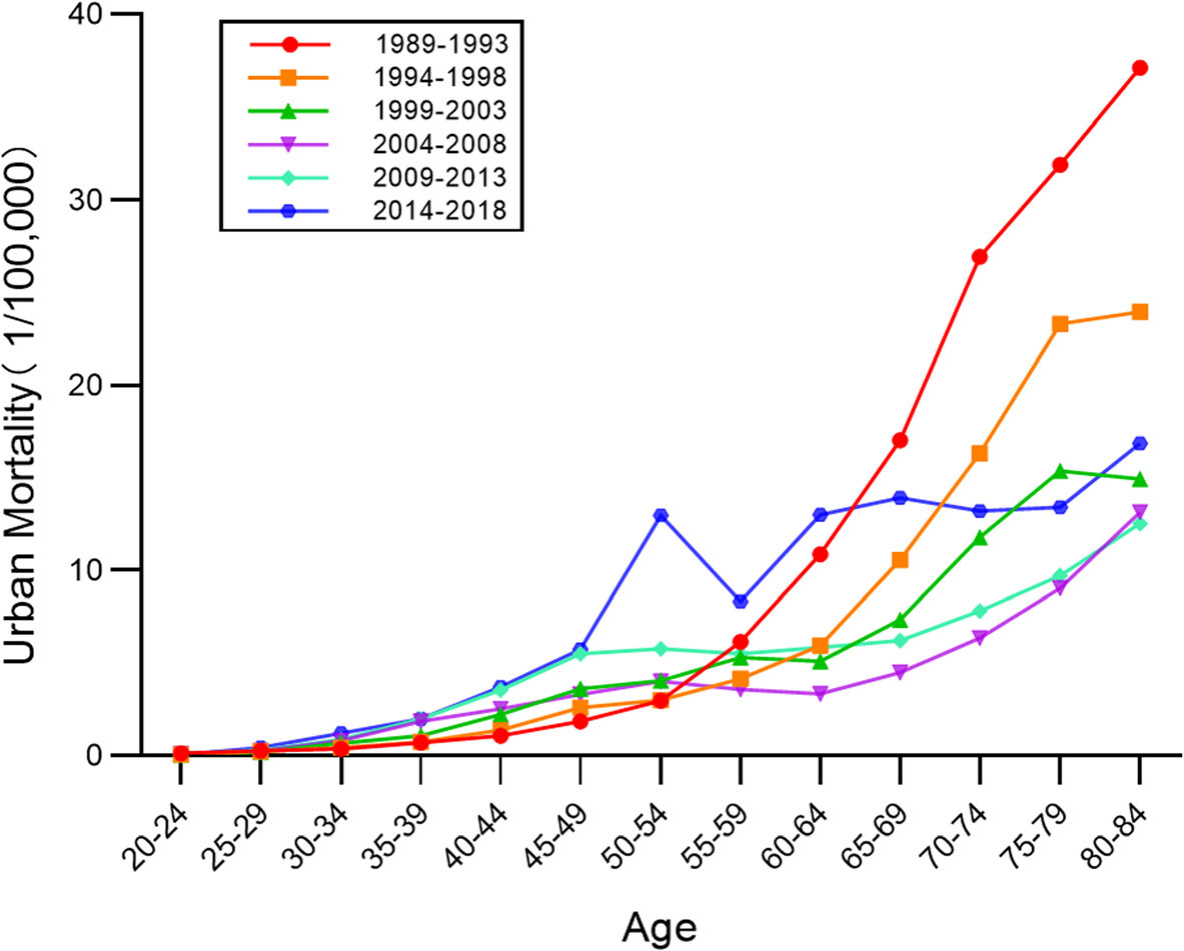
Age-specific mortality rates for cervical cancer in different periods in urban China

**Fig. 4:**
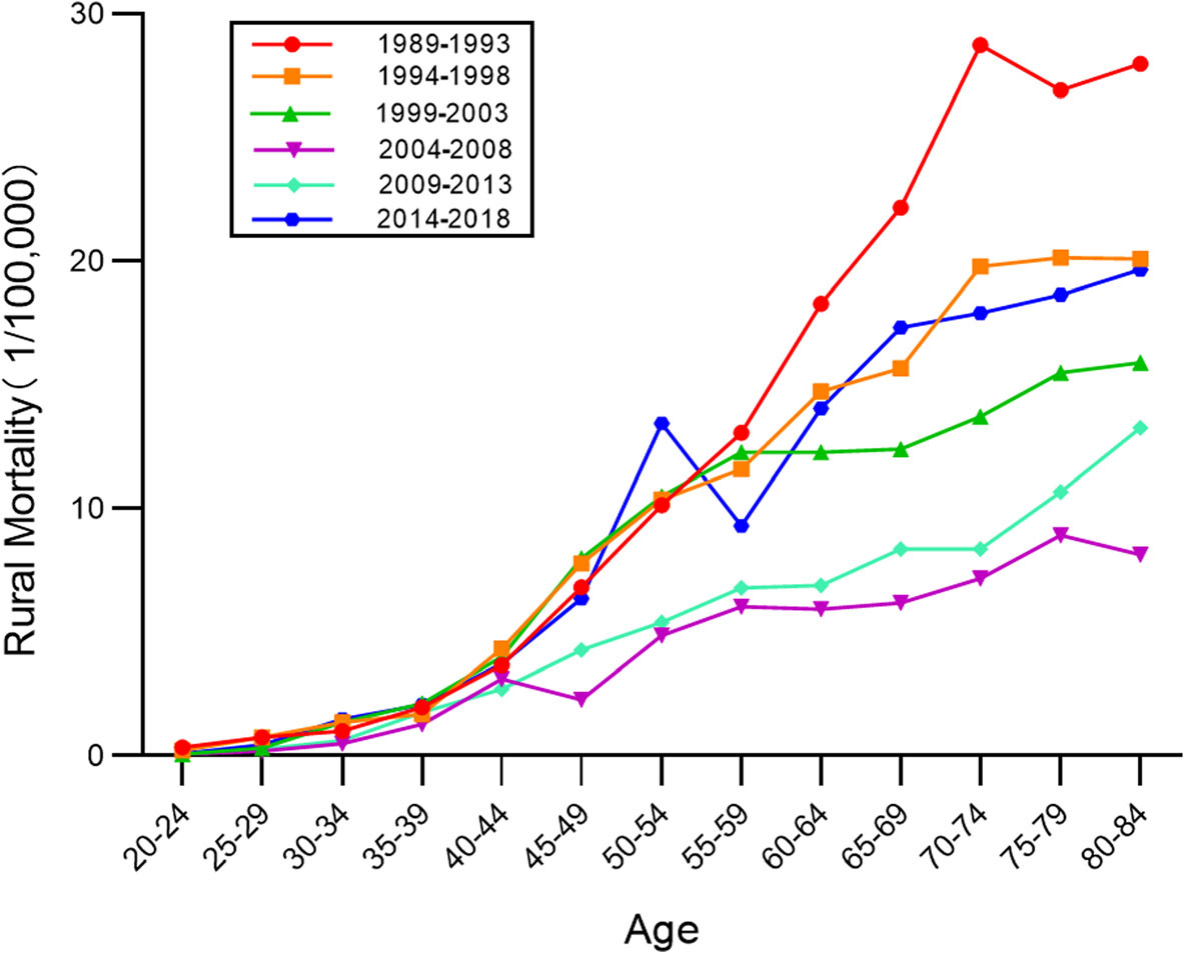
Age-specific mortality rates for cervical cancer in different periods in rural China

Age-specific mortality rates for cervical cancer in different periods in urban and rural China are exhibited in Figs. 3 and 4. On the whole, mortality rates increased with age in all periods. In both urban and rural areas mortality increased steadily and then rose more steeply. The mortality rates show an obvious upward trend beginning with the 50–54 age group in urban areas, and the 40–44 age group in rural areas. In both urban and rural areas, the mortality rates in the 2014–2018 period were at their highest levels before 55–59 age group, and then the mortality rates in 1989–1993 period was highest. It is noteworthy that the highest value of mortality rates in 1989–1993 occurred in urban areas rather than in rural areas.

### Period-specific cervical cancer mortality rates by age group

**Fig. 5:**
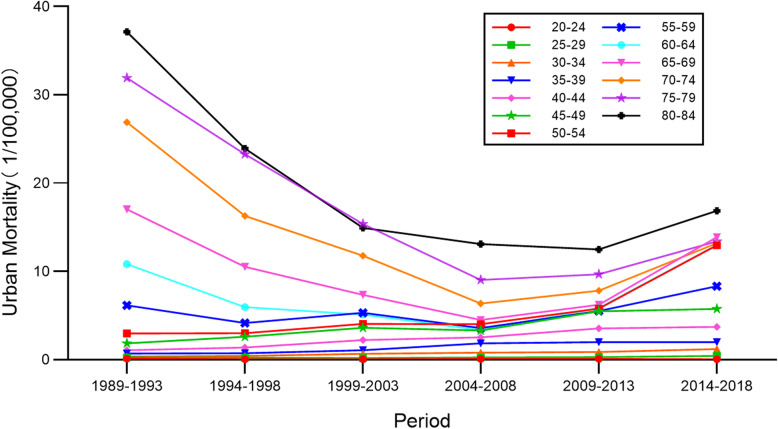
Time trends for cervical cancer mortality rates in different age groups in urban China

**Fig. 6:**
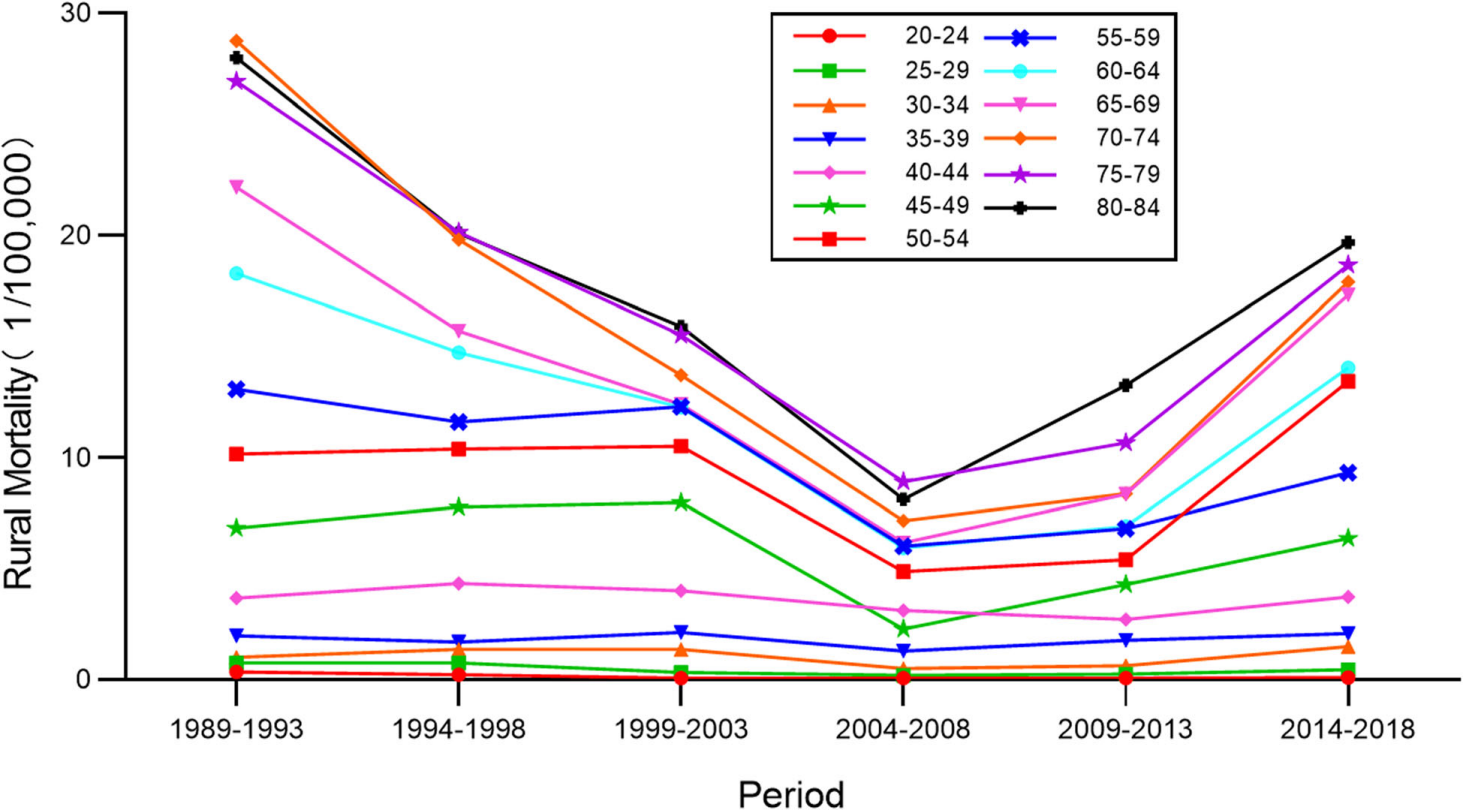
Time trends for cervical cancer mortality rates in different age groups in rural China

Time trends for cervical cancer mortality rates in different age groups in urban and rural China are shown in Figs. 5 and 6. Both rural and urban mortality reached its lowest value in 2004–2008. The older the age group, the higher the mortality rate was. The gap was even greater among the 45–84 years-of-age groups. The largest value in urban areas was higher than it was in rural areas. In urban China, mortality rates for the 65–84 age groups clearly showed a decreasing trend, which then rose upward; there were fluctuations in the 55–64 age groups without an obvious trend; and mortality in the 20–54 age groups increased gradually over the 1989–2018 period. In rural China, mortality rates for the 60–84 age groups first showed decline and then increased gradually; rates in the 20–59 age groups seemed to undulate irregularly during the observed period. Overall, cervical cancer mortality rates exhibited a process of first rising, and then decreasing over the entire observation period.

### Cohort-specific cervical cancer mortality rates by age groups

**Fig. 7:**
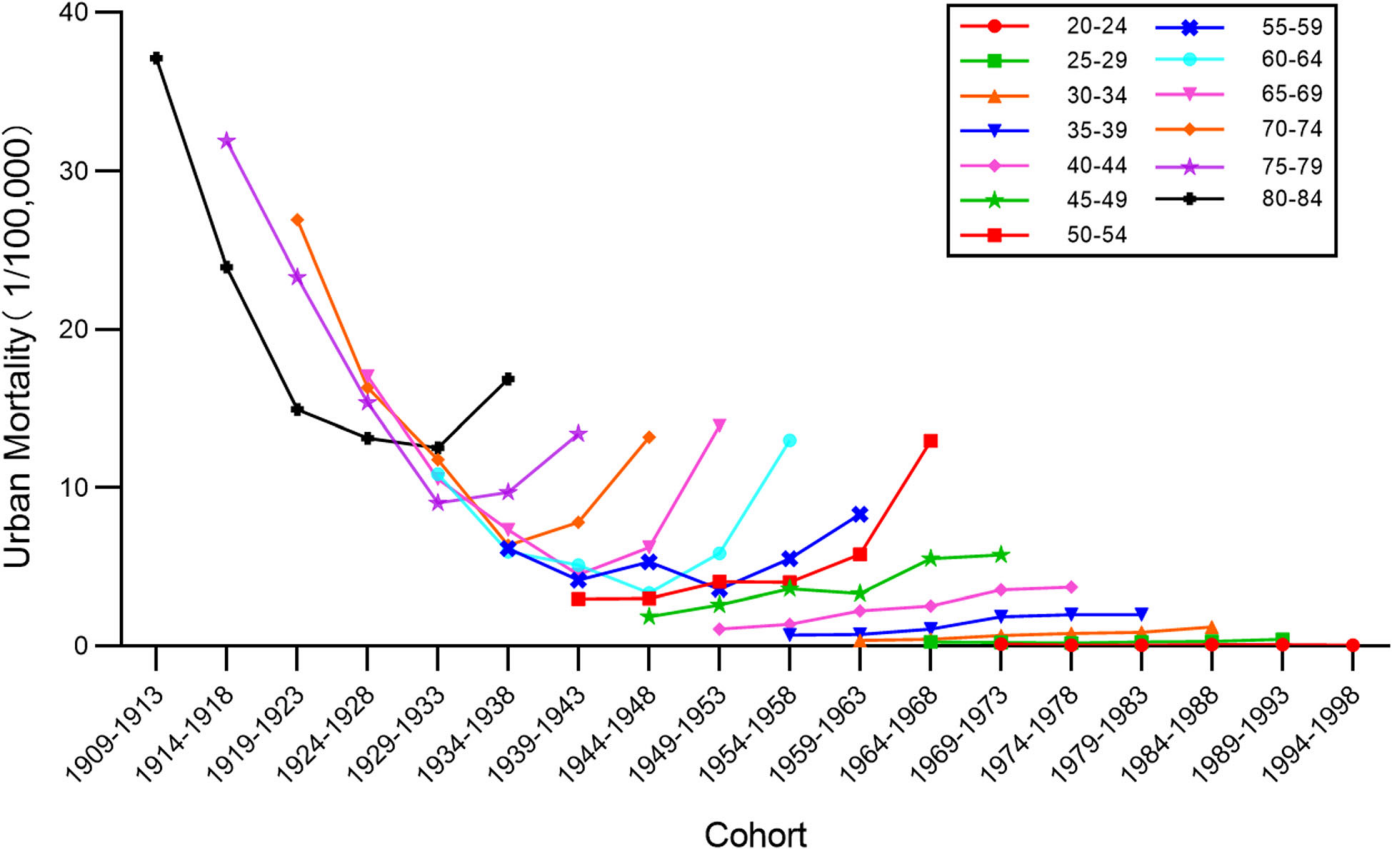
Age-specific mortality rates for cervical cancer mortality rates by birth cohort in urban China

**Fig. 8:**
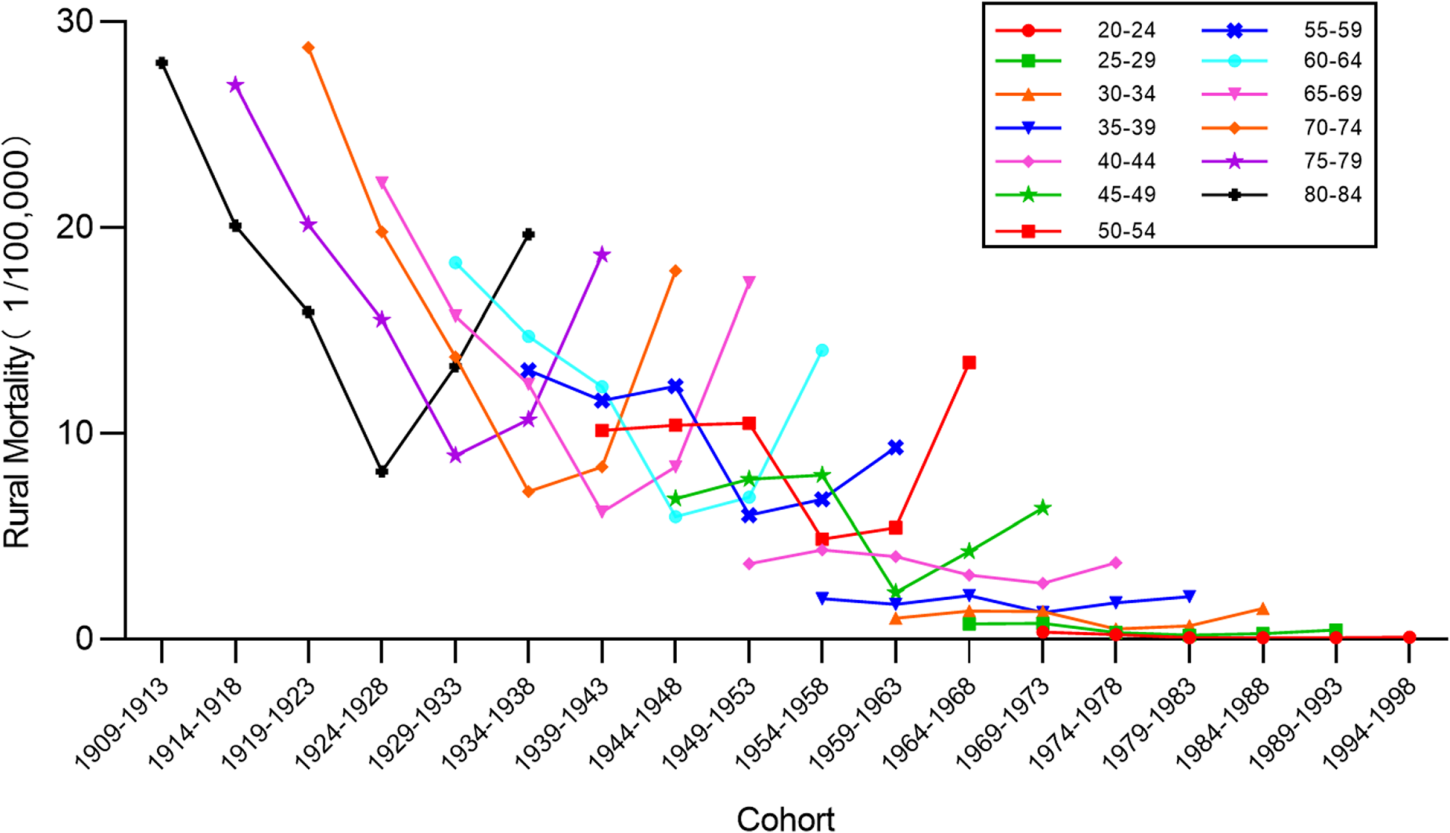
Age-specific mortality rates for cervical cancer mortality rates by birth cohort in rural China

Time trends for cervical cancer mortality rates by birth cohort in urban and rural China are presented in Figs. 7 and 8, respectively. The results indicate that in the same age group, there were different death rates in different birth cohorts. In both urban and rural areas, the mortality rates for the 50–84 age groups declined with the birth cohort and increased thereafter, while rates in the 20–49 age groups fluctuated without any obvious trend. Additionally, mortality rates varied widely among different age groups within the same birth cohort, with rates increasing with age. This finding was more evident in older age groups. It should be noted that the largest value of mortality rate was higher in urban areas than in rural areas.

## Results of APC model

**Fig. 9:**
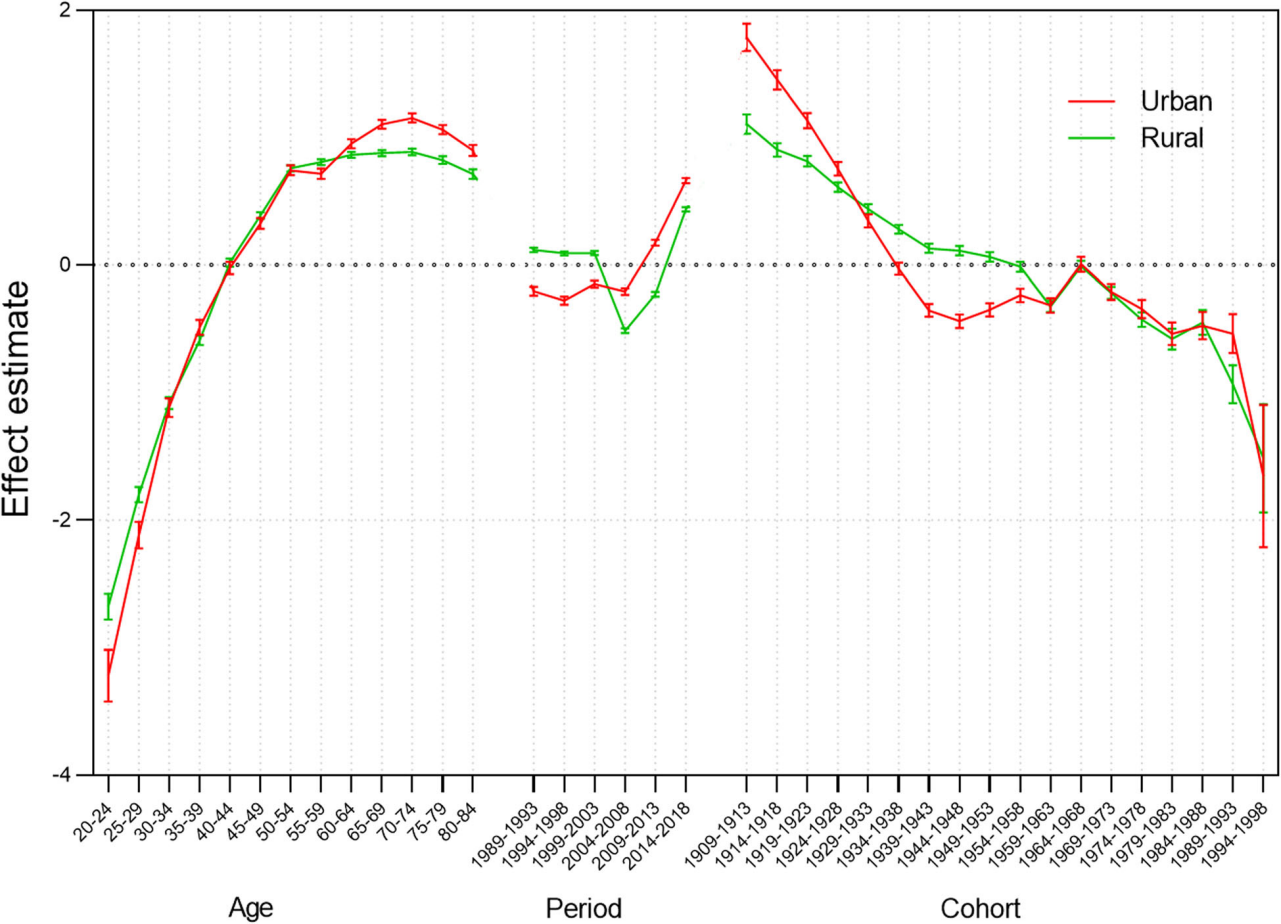
Age, period, and cohort effects on cervical cancer mortality in urban and rural China

The AIC of the APC model for urban and rural China is 19.17 and 23.06 and for the BIC, 648.336 and 892.027, respectively. Estimates of the effects of age, period and cohort, and corresponding confidence interval (95%) are described in Table 3 in Appendix; corresponding trends are shown in Fig. 9. The results suggest that mortality due to cervical cancer is significantly influenced by age, period, and cohort. The variation trend of the three effects indicates that the age effect and cohort effect were the main factors for cervical cancer death risk in Chinese women, while the period effect was relatively less impactful. The respective effects of age, period, and cohort are now further analyzed.

### Age effect

Overall trends in the age effect on cervical cancer mortality risk both in urban and rural areas were similar. The risk of death from cervical cancer increased with age, with the risk increasing dramatically before 54 years of age, and then plateauing. Mortality risk peaked in the 70–74 age group in both urban and rural areas, and the peak value was higher in urban than rural areas. Under the premise of controlling for period and cohort effects, the age effect was a protective factor for the risk of death from cervical cancer for women aged 20–44 years, but a risk factor for women over the age of 45 years. It is worth noting, however, that the estimate of effect in the 40–44 age group was not statistically significant either in rural or urban areas.

### Period effect

For period effect, all estimates were statistically significant. The risk of death from cervical cancer increased after fluctuating. While higher in rural areas before 2003, this risk subsequently reversed. It is remarkable that the period effect had relatively less influence on mortality than either age or cohort effects, since its value was around 0.

### Cohort effect

Contrary to age effect, birth cohort had a negative effect on risk of death from cervical cancer. In the cohorts born earlier, a more dramatic reduction in risk was observed in urban areas than occurred in rural areas. In both areas, the downward trend, which increased slightly before leveling off again, was similar for women born after 1959. Controlling for age and period effects, the cohort effect would increase the risk for cervical cancer death among cohorts born earlier but would reduce it for cohorts born later. Nonetheless, it is notable that the effect estimates for women born between 1934 and 1938 in urban areas, between 1954 and 1958 in rural areas, and in both areas between 1964 and 1968 were not statistically significant.

## Discussion

We conducted a nationwide analysis of trends over time in mortality from cervical cancer among Chinese women using the Joinpoint and APC models to explore the risk factors for cervical cancer mortality. The results of our study show an overall trend of cervical cancer mortality rates initially decreasing, and then increasing over the 30-year period between 1989 and 2018. The upward trend is even more pronounced after 2013. Age and cohort were found to be the main influencing factors that women aged over 50 had contributed a lot to the increase in cervical cancer mortality. This finding serves as a reminder that effective prevention and control measures are required to avert the continuing high numbers of cervical cancer deaths. Further analysis of our findings followed.

The effects of age on cervical cancer mortality was a response to the impact of biological factors associated with aging. It could be inferred from the results of our study that women’s risk of death from cervical cancer increased with growth of age. As a result, the mortality rates of older women were higher than those of younger women. The age effect rose to a positive value for women after the age of 45 years across China, as Fig. 9 showed. This meant that for women over 45 years, age became a factor contributing to the risk of death from cervical cancer. It also meant that women in China over 45-years-old were at a relatively high risk of death from cervical cancer. This might be due to lower participation in prevention programs, and diminished cure rates in older women. This finding was consistent with the study conducted by Bao and Yu [18, 19], which emphasized the lack of awareness about cervical cancer, with women over 50 years-old proving to have significantly lower screening rates than women in the 35–49 age group. That study also stated that failure to treat in time could result in advanced cervical cancer, and death in later life. In cervical cancer, it should be noted, precancerous lesions may exist for a long period of time before diagnosis. Studies have shown that it may take 15 to 20 years from HPV infection to cervical cancer [4], which indicates that women aged 25–30 years might have a relatively higher risk of HPV infection. This conclusion is consistent with many published studies [20, 21]. Therefore, screening and prevention programs targeting this age group should be reinforced.

The period effect reflected the influencing factors of cervical cancer mortality rates caused by a complex set of historical events such as military conflicts, public health interventions, socio-economic development, advances in treatment, and so on. With the passage of time over the observation period, the estimates of period effect fluctuated around the value of 0. This suggested that the period effect had relatively less influence on mortality than did age or cohort. This could be due to the relatively short term of observation. According to detailed analysis, the period effect declined slightly between 1989 and 2008 and rose again between 2009 and 2018. Correspondingly, as shown in Figs. 5 and 6, the age-specific mortality rates in both urban and rural China first decreased, and then rose, especially among rural older age groups. From the perspective of social development, due to China’s massive urbanization and economic development, medical technology and people’s living standards have greatly improved [22, 23]. Up until 2008, the mortality rate was showing a downward trend. In 2009, China launched its NCCSPRA to provide free cervical cancer screening services for registered rural women aged 35–64 years old. Many locally organized screening projects financed by municipal authorities began at the same time. Many potential patients, or patients with comorbidities, were screened and diagnosed with cervical cancer. The IARC database shows that the incidence of cervical cancer in women aged 35–64 has increased significantly in recent years. Correspondingly, mortality has increased with incidence, particularly among elderly women, indicating that newly diagnosed patients were at an advanced stage of cervical cancer or were not receiving appropriate treatment.

The cohort effect explained the influence of different risk exposure on cervical cancer mortality rates caused by different cohorts. Contrary to the age effect, the estimates of cohort effect declined over the observation period. Following a dramatic reduction, estimates of the cohort effect in both urban and rural China have successively decreased to a negative value. This indicates that women born later were less likely to die of cervical cancer. This might be explained by the fact that women born before the 1950s lived in a poor conditions which increased their likelihood of exposure to the risk factors for cervical cancer. Since the founding of new China, living conditions, education and awareness have greatly improved for much of the population [24]. As with the age effect, the long course of cervical cancer is an important influencing factor. Later-born patients with cervical cancer may not have died. It should, however, be noted that in the same birth cohort, mortality rates at first decreased, and then increased with the passage of time. It can be seen from Figs. 7 and 8 that this phenomenon was more pronounced in the early birth cohort, particularly in rural areas. This also shows the elderly were at higher risk of death from cervical cancer, as with the age effect and the period effect.

From the results of age, period, and cohort, it can be concluded that cervical cancer mortality in China has increased in recent years, and particularly after 2013, when the rate of increase accelerated. We found that the age and cohort effects had an important role in the contribution of women aged over 50 years to the increase in cervical cancer mortality. This conclusion differs from Wang [17], possibly owing to the implementation of the NCCSPRA to identify patients who potentially have cervical cancer but have either not received treatment, or treatment was untimely and thus, ineffective. We further explored the causes of the rising mortality rate of women aged over 50, and found three possible reasons. First, limited coverage of cervical cancer screening might prevent some early-stage patients from receiving timely diagnosis and treatment [25, 26]. In rural China, only 10 million women are screened free of charge each year. Given the huge population of China, it would take a relatively long period to realize the benefits of screening [27]. Further, studies have shown that 74.87% of Chinese women think there is no need for screening if they are asymptomatic [28]. Elderly women in particular have inadequate knowledge about cervical cancer [29]. Limited awareness of cervical cancer among rural women has also led to lower screening coverage. Second, the cost of treating advanced cervical cancer due to failure to undertake early treatment can reach more than 100,000 yuan, which is a great financial burden to individuals and families. Faced with high costs, patients may choose to abandon treatment or adopt only palliative care [30], a situation that is more common among elderly women. Third, the high mortality rates among the elderly might be related to more complications in this cohort, reducing the survival rates of patients with cervical cancer as the main cause.

In response to the issues discussed above, we propose the following suggestions. First, the coverage of screening should be expanded. Where resources are limited, priority should be given to high-risk groups, such as women living with HIV, immunosuppressed women, and so on [31]. Also, the coverage of once-in-a-lifetime preventive screening for eligible women should be increased. Second, research has demonstrated that improving the target population’s awareness of cervical cancer could increase the numbers willing to be screened [32]. Therefore, raising awareness of cervical cancer among women, especially older women, is essential to expanding screening coverage. Third, only timely treatment following a positive result from screening can prevent advanced cervical cancer and potential death, and ultimately reduce mortality rates. To avoid the high costs of treating advanced cervical cancer, early diagnosis and early treatment is critical. The IARC database shows that the incidence of cervical cancer in women aged 35 to 64 years has increased significantly in recent years [2]. Therefore, attention should be given to the treatment of this group to prevent an increase in the mortality rates of elderly women in the future. In addition, the HPV vaccine had been accessible in parts of China since 2016 [33]. It has shown a positive effect on the prevention of HPV infection among the Chinese urban population. However, due to its high cost, limited quantity and length of time to market, it will take considerable time before certain benefits can be realized. Therefore, cervical cancer screening currently remains the main method to prevent cervical cancer in China. Timely treatment is the main means to reduce cervical cancer mortality.

A major limitation of this study is that the analysis of cervical cancer incidence was not provided, because the incidence period (1998–2012) in the IARC database did not match the mortality period in this study. Another reason for this omission is that many patients would be diagnosed with the gradual expansion of screening coverage in China. Therefore, the incidence rates might continue to rise in the future for a period. After 2009, the incidence of women aged 35 to 64 years began to rise, which proved this conclusion. But the purpose of cervical cancer screening is to find more potential cervical cancer patients and cure them, thereby reducing the mortality rate of cervical cancer. On this count, we did a targeted analysis of mortality. Another limitation is that China experienced massive urbanization during the study’s observation period, and changes in the size of rural and urban populations might have had some impact on the mortality data.

## Conclusion

In summary, we found that across rural and urban parts of China, mortality rates from cervical cancer initially declined, but then increased over the entire observation period. Utilizing the APC model, we found that age and cohort had significant effects on cervical cancer mortality, but in opposite directions. For women over the age of 45 years, age became a contributing factor to the risk of death from cervical cancer. The cohort effect became a protective factor for women born after 1938 in urban areas, and for women born after 1958 in rural areas. The period effect had relatively less influence on mortality than did age and cohort effects. This result suggests that attention to cervical cancer screening and treatment for older women is warranted.

## Data Availability

All data is freely available and open for public perusal. Accessed June 22, 2021. If someone wants to request the data from this study, also can contact the corresponding author by email xujuan@hust.edu.cn.

## Appendix 1

**Table 1:** Age-specific mortality rates (per 100,000 women) of cervical cancer in urban China

Age	Year of Death					
	1989–1993	1994–1998	1999–2003	2004–2008	2009–2013	2014–2018
20–24	0.12	0.06	0.04	0.09	0.07	0.05
25–29	0.24	0.21	0.18	0.26	0.28	0.44
30–34	0.36	0.44	0.66	0.81	0.87	1.20
35–39	0.68	0.74	1.07	1.86	1.97	1.97
40–44	1.06	1.38	2.22	2.53	3.54	3.71
45–49	1.84	2.60	3.61	3.31	5.49	5.73
50–54	2.98	3.00	4.06	4.01	5.78	12.97
55–59	6.14	4.14	5.29	3.57	5.51	8.32
60–64	10.84	5.96	5.09	3.35	5.85	13.00
65–69	17.02	10.54	7.33	4.50	6.22	13.89
70–74	26.90	16.30	11.76	6.36	7.82	13.19
75–79	31.90	23.28	15.37	9.03	9.69	13.40
80–84	37.12	23.92	14.92	13.11	12.50	16.85

**Table 2:** Age-specific mortality rates (per 100,000 women) of cervical cancer in rural China

Age	Year of Death					
	1989–1993	1994–1998	1999–2003	2004–2008	2009–2013	2014–2018
20–24	0.35	0.21	0.07	0.07	0.05	0.09
25–29	0.75	0.75	0.32	0.20	0.25	0.44
30–34	1.01	1.35	1.35	0.49	0.63	1.48
35–39	1.97	1.70	2.12	1.29	1.76	2.07
40–44	3.66	4.33	4.00	3.10	2.71	3.71
45–49	6.82	7.77	7.97	2.27	4.27	6.36
50–54	10.14	10.38	10.49	4.87	5.40	13.43
55–59	13.07	11.59	12.28	6.02	6.79	9.31
60–64	18.29	14.72	12.26	5.93	6.89	14.04
65–69	22.17	15.68	12.39	6.17	8.35	17.32
70–74	28.75	19.80	13.71	7.16	8.37	17.90
75–79	26.92	20.13	15.50	8.91	10.66	18.65
80–84	27.98	20.09	15.89	8.13	13.25	19.66

## Appendix 2

**Table 3:** APC model analysis results of Chinese female cervical cancer mortality

Factor	Urban			Rural		
	Coef.	95% CI	*P*-value	Coef.	95% CI	*P*-value
**Age (years)**						
20–24	−3.219	(−3.421, −3.017)	0.000	−2.679	(−2.780, −2.578)	0.000
25–29	−2.117	(−2.221, −2.013)	0.000	−1.799	(−1.859, −1.740)	0.000
30–34	−1.118	(−1.191, −1.045)	0.000	−1.082	(−1.128, −1.035)	0.000
35–39	−0.486	(− 0.545, − 0.428)	0.000	− 0.589	(− 0.627, − 0.551)	0.000
40–44	− 0.025	(− 0.074, 0.024)	0.323	0.019	(− 0.012, 0.050)	0.224
45–49	0.326	(0.283, 0.369)	0.000	0.389	(0.363, 0.416)	0.000
50–54	0.744	(0.705, 0.784)	0.000	0.759	(0.734, 0.783)	0.000
55–59	0.717	(0.678, 0.757)	0.000	0.809	(0.785, 0.833)	0.000
60–64	0.953	(0.917, 0.989)	0.000	0.865	(0.842, 0.888)	0.000
65–69	1.106	(1.071, 1.141)	0.000	0.879	(0.855, 0.903)	0.000
70–74	1.155	(1.121, 1.190)	0.000	0.890	(0.864, 0.915)	0.000
75–79	1.063	(1.028, 1.099)	0.000	0.824	(0.795, 0.854)	0.000
80–84	0.900	(0.858, 0.942)	0.000	0.715	(0.677, 0.753)	0.000
**Period**						
1989–1993	− 0.204	(− 0.239, − 0.169)	0.000	0.118	(0.101, 0.136)	0.000
1994–1998	− 0.279	(− 0.310, − 0.248)	0.000	0.092	(0.076, 0.108)	0.000
1999–2003	−0.147	(−0.175, − 0.120)	0.000	0.094	(0.078, 0.110)	0.000
2004–2008	−0.207	(−0.233, − 0.182)	0.000	−0.514	(− 0.533, − 0.494)	0.000
2009–2013	0.176	(0.153, 0.198)	0.000	−0.229	(−0.248, − 0.210)	0.000
2014–2018	0.662	(0.642, 0.683)	0.000	0.438	(0.421, 0.455)	0.000
**Cohort**						
1909–1913	1.786	(1.678, 1.894)	0.000	1.107	(1.031, 1.183)	0.000
1914–1918	1.453	(1.378, 1.528)	0.000	0.905	(0.853, 0.956)	0.000
1919–1923	1.133	(1.073, 1.193)	0.000	0.815	(0.774, 0.856)	0.000
1924–1928	0.756	(0.703, 0.810)	0.000	0.612	(0.575,0.650)	0.000
1929–1933	0.347	(0.296, 0.397)	0.000	0.441	(0.405, 0.477)	0.000
1934–1938	−0.029	(−0.077, 0.018)	0.227	0.281	(0.247, 0.316)	0.000
1939–1943	−0.354	(−0.404, − 0.303)	0.000	0.131	(0.096, 0.166)	0.000
1944–1948	−0.439	(−0.491, − 0.387)	0.000	0.113	(0.077, 0.149)	0.000
1949–1953	−0.350	(−0.402, − 0.298)	0.000	0.065	(0.028, 0.102)	0.001
1954–1958	−0.236	(−0.289, − 0.183)	0.000	−0.014	(− 0.052, 0.024)	0.477
1959–1963	−0.315	(− 0.373, − 0.257)	0.000	−0.323	(− 0.366, − 0.279)	0.000
1964–1968	0.006	(− 0.052, 0.064)	0.834	− 0.009	(− 0.052, 0.034)	0.675
1969–1973	− 0.210	(− 0.273, − 0.147)	0.000	−0.218	(− 0.265, − 0.171)	0.000
1974–1978	−0.343	(− 0.415, − 0.272)	0.000	−0.427	(− 0.485, − 0.369)	0.000
1979–1983	−0.538	(− 0.626, − 0.449)	0.000	−0.579	(− 0.660, − 0.499)	0.000
1984–1988	−0.475	(− 0.582, − 0.368)	0.000	−0.449	(− 0.546, − 0.353)	0.000
1989–1993	−0.537	(− 0.688, − 0.385)	0.000	−0.935	(−1.083, − 0.787)	0.000
1994–1998	−1.655	(−2.213, − 1.097)	0.000	− 1.515	(− 1.940, − 1.091)	0.000
Cons	−10.381	(− 10.416, -10.346)	0.000	− 10.121	(− 10.149, − 10.093)	0.000
**Model fitting**						
	AIC	19.168		AIC	23.064	
	BIC	648.336		BIC	892.027	

## Abbreviations

DALY: Disability adjusted life year
APC: Age-period-cohort
IE: Intrinsic estimator
IARC: The International Agency for Research on Cancer
WHO: The World Health Organization
HPV: Human papillomavirus
ASMR: The age-standardized mortality rate
NCCSPRA: The National Cervical Cancer Screening Program in Rural Areas
JRP: The Joinpoint Regression Program
AIC: The Akaike Information Criterion
BIC: The Bayesian Information Criterion
CSCCP: The Chinese Society for Colposcopy and Cervical Pathology of China Healthy Birth Science Association
